# H3.3 kinetics predicts chromatin compaction status of parental genomes in early embryos

**DOI:** 10.1186/s12958-021-00776-3

**Published:** 2021-06-11

**Authors:** Shi-meng Guo, Xing-ping Liu, Li-quan Zhou

**Affiliations:** Institute of Reproductive Health, Tongji Medical College, Huazhong University of Science and Technology, 430030 Wuhan, Hubei China

**Keywords:** H3.3, Chromatin mobility, Totipotency

## Abstract

**Background:**

After fertilization, the fusion of gametes results in the formation of totipotent zygote. During sperm-egg fusion, maternal factors participate in parental chromatin remodeling. H3.3 is a histone H3 variant that plays essential roles in mouse embryogenesis.

**Methods:**

Here, we used transgenic early embryos expressing H3.3-eGFP or H2B-mCherry to elucidate changes of histone mobility.

**Results:**

We used FRAP analysis to identify that maternally stored H3.3 has a more significant change than H2B during maternal-to-embryonic transition. We also found that H3.3 mobile fraction, which may be regulated by *de novo* H3.3 incorporation, reflects chromatin compaction of parental genomes in GV oocytes and early embryos.

**Conclusions:**

Our results show that H3.3 kinetics in GV oocytes and early embryos is highly correlated with chromatin compaction status of parental genomes, indicating critical roles of H3.3 in higher-order chromatin organization.

**Supplementary Information:**

The online version contains supplementary material available at 10.1186/s12958-021-00776-3.

## Background

Embryo development begins with fusion of terminally differentiated gametes. The maternal factors stored in the oocytes are involved in epigenetic reprogramming of genome. The fusion of the gametes upon fertilization results in the formation of a totipotent cell which experiences totipotency-to-pluripotency transition, and finally forms different cell lineages. This process involves multiple reprogramming events, including chromatin reassembly [1, 2], the binding of histone variants, histone modification [3, 4], and so on.

Nucleosomes, composed of octamers of histones, are basic components of chromatin structures in cells. Histone variants and histone modifications provide nucleosomes with increased dimensions to orchestrate complex events including chromatin assembly, epigenetic remodeling and gene transcription. Recently, it was discovered that prenucleosomes composed of histone tetramers are stable isomers of canonical nucleosomes [5]. Prenucleosomes can be formed within seconds and converted to canonical nucleosomes in an ATP-dependent way. Several unique features of chromatin structures in early mouse embryos have been documented but detailed mechanism remain enigmatic, and uncovering non-canonical nucleosome structures like prenucleosomes may facilitate understanding of formation and function of special chromatin structures in early embryos.

Nucleosome assembly can take place in a replication-dependent or -independent manner. While the canonical histones are deposited in a replication-dependent manner, the histone H3 variant H3.3 can be deposited in a replication-independent manner [6, 7]. Studies have shown that histone variant H3.3 is the main H3 protein on the female chromatin in oocytes [8]. H3.3 was reported to be involved in chromatin reprogramming [9], and incorporate into chromatin in a DNA replication-independent manner during the oocyte-to-embryo transition [8, 10]. During oogenesis, maternal H3.3 deficiency leads to cell death [11]. H3.3 is necessary for formation of male pronucleus, with maternal H3.3 depletion resulting in pronucleus forms a nuclear envelope devoid of nuclear pore complexes (NPCs) [12]. During the early embryo development, H3.3 regulates efficient assembly of pericentric heterochromatin [13]. H3.3 also maintains the decondensation state of early embryonic chromatin by antagonizing histone H1, and regulates expression of pluripotency factors in early embryos [14].

Fluorescence recovery after photobleaching (FRAP) is a valuable tool to study the mobility of histones in oocytes/embryos [15]. This technique reveals the the extent of chromatin looseness by detecting the histone mobility in nuclei [15, 16]. In this study, we used transgenic female mice expressing H3.3-eGFP and H2B-mCherry in oocytes and generated early embryos with wildtype male for histone mobility analysis by FRAP. We also analyzed the relationship between maternal H3.3 mobility and chromatin compaction of parental genomes in oocytes and early embryos.

## Methods

### Animals

Mice were all housed in pathogen-free facility of Huazhong University of Science and Technology. All mice used in this study were maintained in FVB background. pCAG-H3.3^eGFP^ transgenic mice (transgene was driven by CAG promoter) and ZP3-H2B^mCherry^ transgenic mice (transgene was driven by ZP3 promoter) had normal fertility (Fig. S1).

### Oocyte/embryo collection and immunostaining examination

Generally, wildtype female mice, pCAG-H3.3^eGFP^ transgenic female mice and ZP3-H2B^mCherry^ transgenic female mice were superovulated to collect GV oocytes from ovaries or mated with wildtype male to collect zygotes. Oocytes were cultured in M16 (Sigma) medium with 2.5µM milrinone (Millipore). Embryos were cultured in KSOM (Millipore) medium. For immunostaining examination, embryos were fixed and permeabilized with 4 % paraformaldehyde and 0.5 % Triton in PBS for 30 min. Embryos were then washed for 3 times in PBST (0.05 % Tween in PBS), blocked for 30 min and incubated with the primary antibodies overnight at 4 °C, followed by 3 washes in PBST and incubation for 2 h with the secondary antibodies and Hoechst 33,342 (10 µg/ml). The primary antibodies used include anti-H3K4ac (Abclonal, A16078) and anti-H3K27ac (Abcam, ab4729). Embryos were observed under LSM 780 confocal microscope.

### FRAP analysis

FRAP was performed using a LSM 780 confocal microscope. Oocytes or embryos were placed in a glass-bottomed dish. Two pre-bleaching frames were acquired, and then a round area of 4 µm^2^ was bleached, followed by acquisition of subsequent images (0.5 frame s-1) for at least 90 s. The raw data was done on background-subtracted and further divided by the intensity of the whole nucleus measured in the corresponding frame. The obtained curves were normalized using the full-scale normalization method so that the first post-bleach frame was set to 0. Normalized curves were then subjected to curve fitting. We distinguished between male and female genome based on the size of the pronucleus because the male pronucleus is always bigger than the female pronucleus (Fig. S2).

### Microinjection followed by fixation and examination

H3.3-mCherry cRNA was produced using T7 polymerase for *in vitro* mRNA synthesis (mMessage mMachine Kit, poly(A) tailing Kit; Thermo Fisher Scientific) and purified using MEGAclear Kit (Thermo Fisher Scientific). About 10pl of 600 µg/µl cRNA was injected into embryos at indicated time and cultured for 4 h, followed by fixation and permeabilization (4 % paraformaldehyde and 0.5 % Triton X-100 for 30 min at room temperature). Embryos were then washed and counterstained with Hoechst 33,342 (10 µg/ml) for observation under LSM 780 confocal microscope.

### Statistical analysis

SigmaPlot software was used for curve fitting, using a one-phase exponential association equation of Y = YMax(1-e^− KX^) where Y = relative intensity, YMax = mobile fraction, K = association constant, and X = time [17].

## Results

**Fig. 1:**
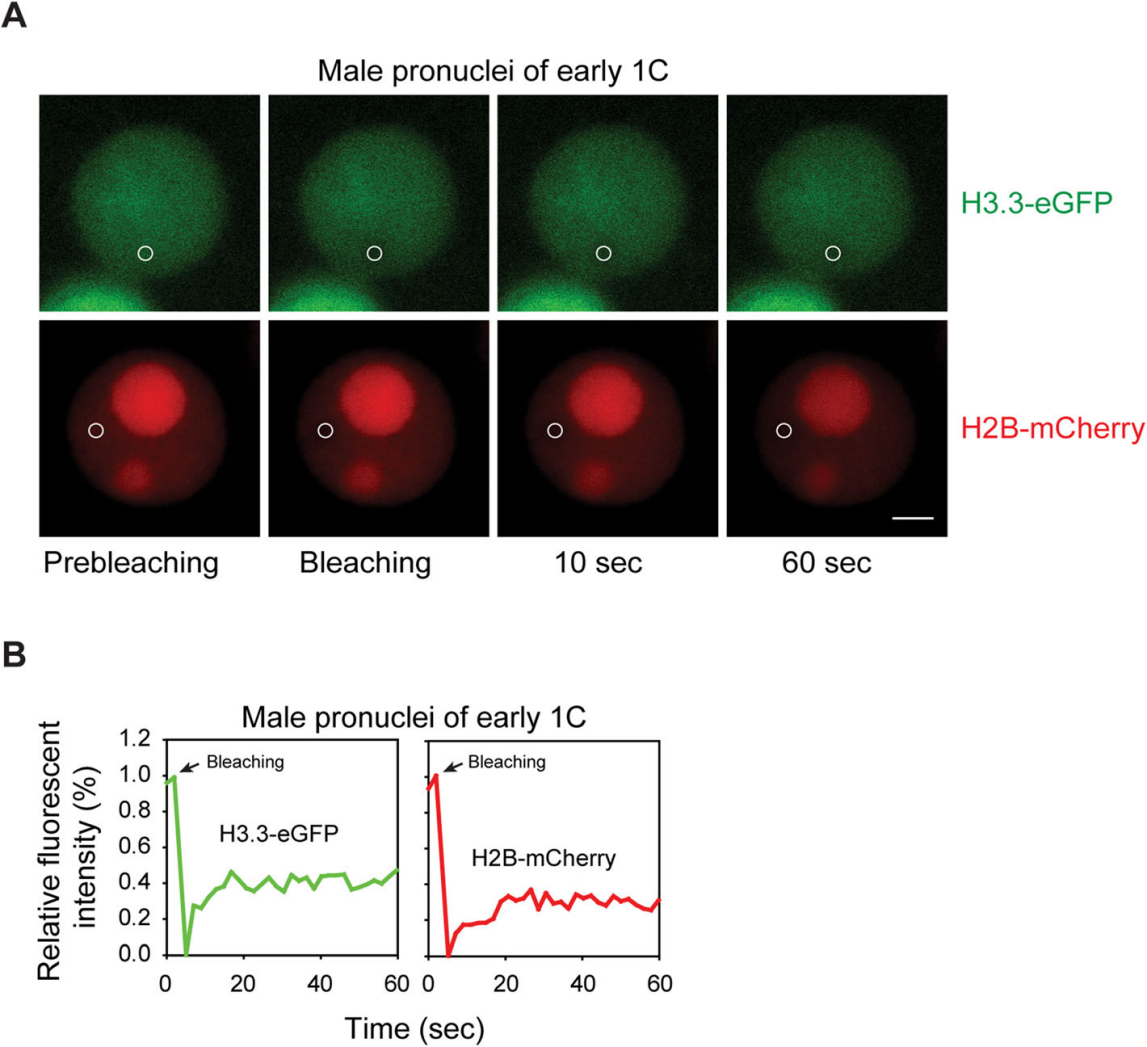
FRAP analysis of maternal histones in early zygote stage.** a** Early zygotes were isolated from H3.3-eGFP and H2B-mCherry transgenic mice and images were collected shortly before and after photobleaching a small area (circle) in male pronuclei. Representative images of prebleaching, bleaching, 10 s after bleaching and 60 s after bleaching. Scale bar, 5 μm.  **b** FRAP recovery curves of maternally expressed H3.3-eGFP and H2B-mCherry in parental genomes of representative early zygotes are displayed

Cleavage-stage embryos have large nuclei relative to cell size compared to later developmental stages which indicates the existence of poorly understood, but unique, chromatin structures. To study the dynamic chromatin packaging of parental genomes, we generated embryos expressing H3.3-eGFP or H2B-mCherry. We performed FRAP examination to assess the mobility of maternal H3.3-eGFP and maternal H2B-mCherry (Fig. 1). FRAP acquisition for H3.3/H2B and single FRAP curves of male pronuclei are shown in Fig. 1a and b, respectively. Non-S phase stages, including GV, early 1 C (G1 phase), late 1 C (G2 phase), early 2 C (G1 phase) and late 2 C (G2 phase), were used to exclude influences from DNA replication.

**Fig. 2:**
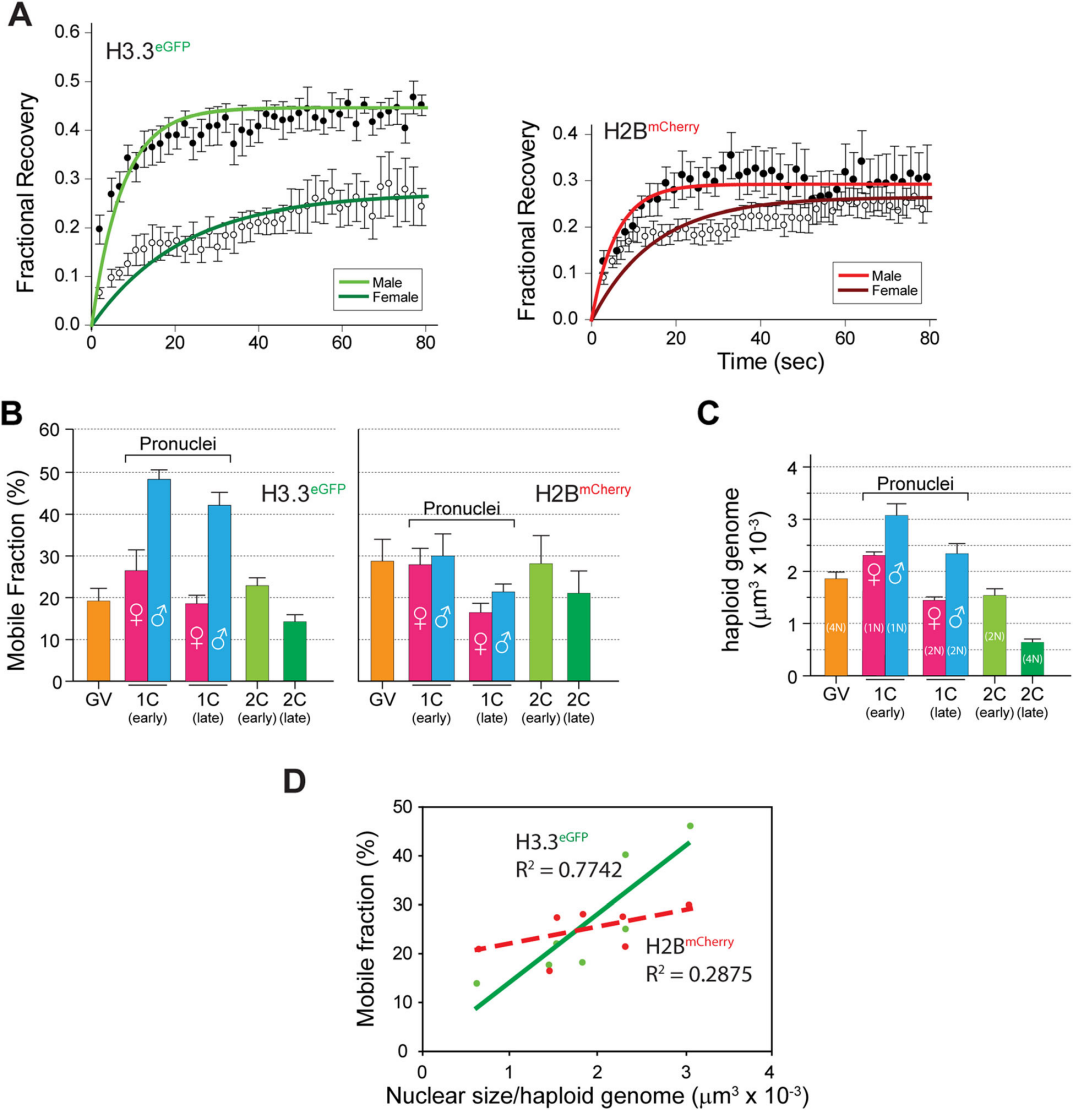
FRAP analysis of maternal histones in GV oocytes and early embryos. **a** FRAP recovery curves of maternally expressed H3.3-eGFP or H2B-mCherry in parental genomes at early zygote stage. Mean ± s.e.m. **b** Compilation of histone mobilities determined by FRAP of maternally expressed H3.3-eGFP (left) and H2B-mCherry (right) in GV oocytes, 1C-early (male), 1C-early (female), 1C-late (male), 1C-late (female), 2C-early and 2C-late embryos. Mean of 8-15 ± s.e.m. **c** Chromatin compaction was determined by nuclear volume per haploid genome during development from GV oocytes to late 2-cell embryos. Mean of 5 ± s.e.m. **d** Linear regression analysis of histone dynamics and nuclear size indicates that mobility of H3.3 instead of H2B correlates linearly with chromatin compaction status

FRAP recovery curves indicated that maternally expressed H3.3 was more different in parental genomes compared with H2B at early zygote stage (Fig. 2a). FRAP curves were used to quantitate mobile fractions, which showed that H2B-mCherry was relatively stable in parental genomes at most stages (Fig. 2b). Interestingly, H3.3-eGFP mobility varies at different stages, with relatively higher mobility in male genome compared to female genome in zygote stage.

Furthermore, we analyzed chromatin compaction status in GV and cleavage embryos to identify its correlation with histone mobility by calculating nuclear size per haploid genome (for example, total nuclear size should be divided by four in GV stage) (Fig. 2c). Notably, our results showed that mobile fraction of H3.3-eGFP had high correlation with chromatin compaction status, while H2B-mCherry mobility seemed irrelevant to chromatin compaction status (Fig. 2d). Our results indicate that H3.3 mobility reflects the compaction status of parental genomes in GV oocytes and early embryos.

**Fig. 3:**
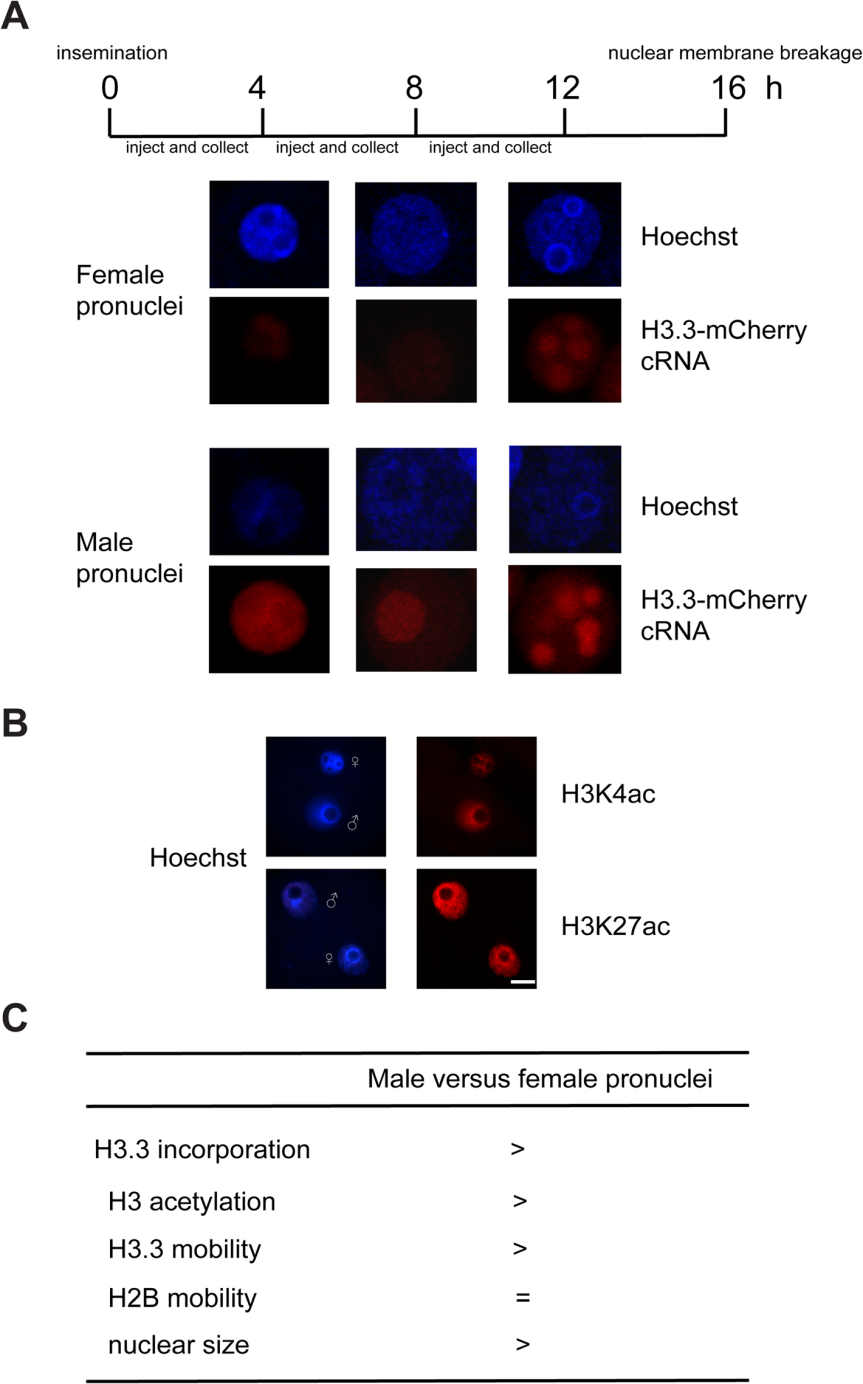
Examination of H3.3 incorporation in early embryos. **a** H3.3-mCherry cRNA was injected at indicated time point and collected for fixation 4 hours later to allow translation and incorporation.**b** Immunostaining against H3K4 acetylation (H3K4ac) and H3K27 acetylation (H3K27ac) was performed using mouse zygotes. Scale bars, 20 μm. **c** Summarization of asymmetry of parental chromatin status

Differential H3.3 mobility may be explained by differential *de novo* H3.3 incorporation because newly synthesized histones for chromatin incorporation are always highly acetylated. We then examined activities of *de novo* H3.3 incorporation by microinjection of H3.3-mCherry cRNA to compare H3.3 incorporation in male and female pronuclei because parental chromatin in zygote showed the most significant differences. Our result showed that male pronuclei consistently had higher *de novo* incorporation of H3.3 than female pronuclei (Fig. 3a). This result is consistent with our observation that H3 acetylation is higher in male pronuclei than female pronuclei (Fig. 3b), facilitating higher transcriptional activity in male pronculei. Therefore, we propose that *de novo* incorporated H3.3 increases global H3.3 mobility to decompact the genome (Fig. 3c).

## Discussion

The chromatin of sperm is mainly wrapped by protamine instead of histone. After gametes fusion, the protamine is gradually replaced by the histone stored in the oocyte, thereby repacking the male chromatin. Histone variant H3.3 is the main H3 protein on the female chromatin in oocyte, which involved in the occurrence of protamine replacement [8, 10]. In this process, the removal of protamine results in male chromatin decondensation. The aggregation of histones on the male chromatin results in male chromatin reaggregation into a symmetrical ellipsoid. With the formation of the nuclear membrane, a large number of nuclear factors import in the nucleus rapidly. Then the ellipsoid chromatin structure expands rapidly and matures into pronucleus [18]. The protamine-histone conversion on the male chromatin is carried out without DNA replication [19]. Classical histones are mainly expressed in the S phase and incorporated into the genome in a replication-coupled manner [20]. Histone variants have homologous sequences and share the similar structure with classic histones [21]. Histone variants expressed throughout the whole cell cycle and incorporated into the genome during embryo development [22]. Histone variants replace classic histones in specific genomic regions to form a nucleosome structure with unique biophysical properties. Histone variants play an important role in chromatin reprogramming and embryonic development.

In our results, maternal H3.3-eGFP displayed a striking, reproducible high mobility in male pronuclei and showed linear correlation with chromatin compaction status, while maternal H2B-mCherry did not. What’s more, H3.3 displayed higher mobility than H2B in male pronucleus. The difference between H3.3 and H2B mobility in parental genomes may be explained by temporary, but prominent, H3.3-H4-DNA structures lacking the H2A-H2B complex. This structure is theoretically unstable and cannot supercoil DNA, possibly resulting in specific higher-order chromatin structure controlling unique transcriptome profiles [23] and high chromatin mobility [3] in these totipotent cells.

## Conclusions

Taken together, we show that the mobility profile of H3.3 corresponds closely to the compaction status of the genome, indicating that H3.3 is involved in higher-order chromatin organization in oocytes and early embryos, probably through mediating unique chromatin structure formation during maternal-to-embryonic transition.

## Supplementary Information

**Additional file 1: Figure S1.** Fertility of wildtype (WT) female mice, pCAG-H3.3^eGFP^ transgenic female mice and ZP3-H2B^mCherry^ transgenic female mice.

**Additional file 2: Figure S2.** Quantification of diameters of male and female pronuclei in zygotes with maternally expressed H3.3-eGFP or H2B-mCherry. ***p<0.001 by Student’s t-test.

## Data Availability

The datasets used and/or analysed during the current study are available from the corresponding author on reasonable request.

## Abbreviations

NPCs: Nuclear pore complexes
FRAP: Fluorescence recovery after photobleaching
