# Supplementary figures and images for: H3.3 kinetics predicts chromatin compaction status of parental genomes in early embryos
Source: Reprod Biol Endocrinol. 2021 Jun 11;19:87. doi: 10.1186/s12958-021-00776-3 (PMC8194155; doi:10.1186/s12958-021-00776-3)

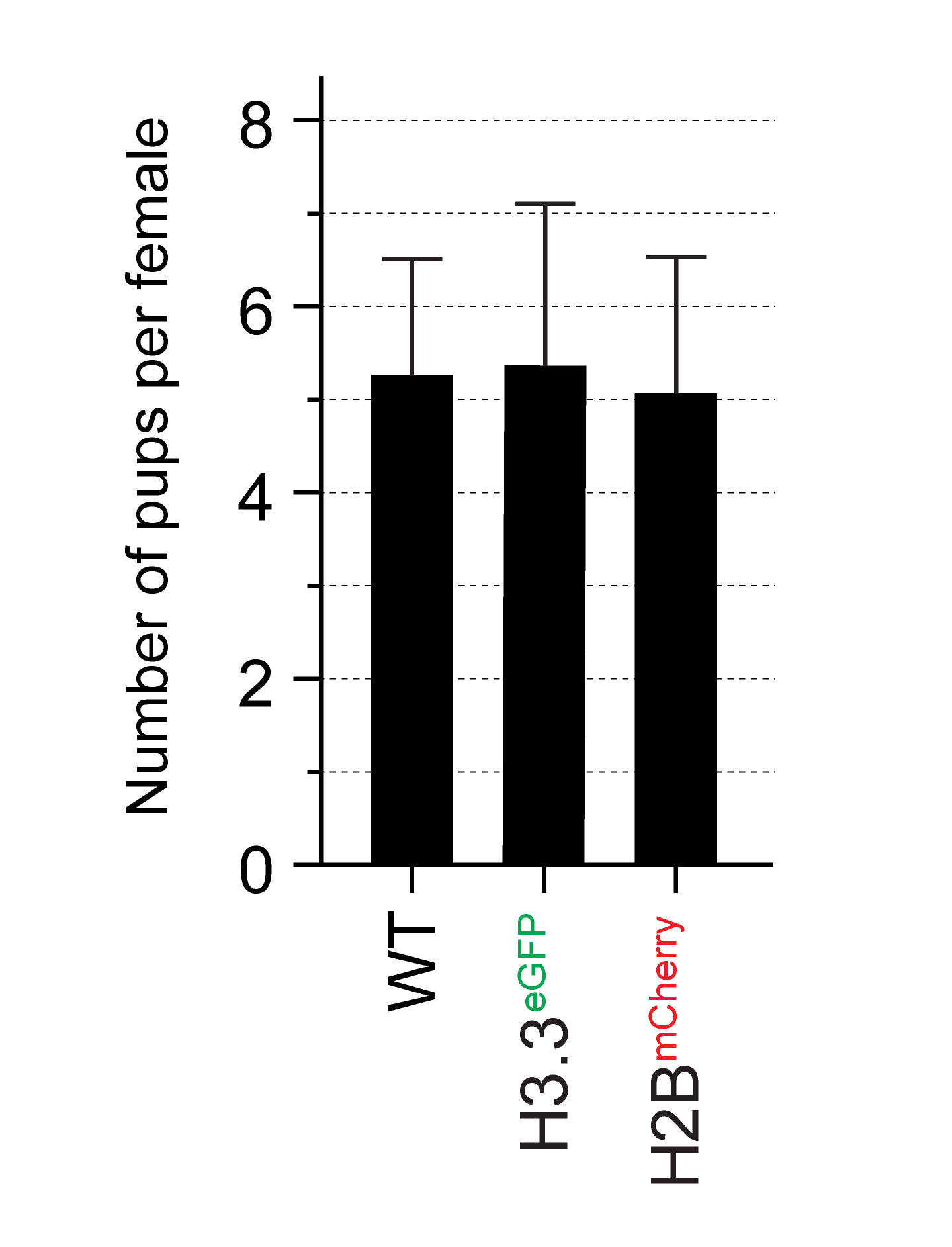

Supplement: Supplementary file 1 — Additional file 1: Figure S1. Fertility of wildtype (WT) female mice, pCAG-H3.3eGFP transgenic female mice and ZP3-H2BmCherry transgenic female mice. [file 12958_2021_776_MOESM1_ESM.tif]

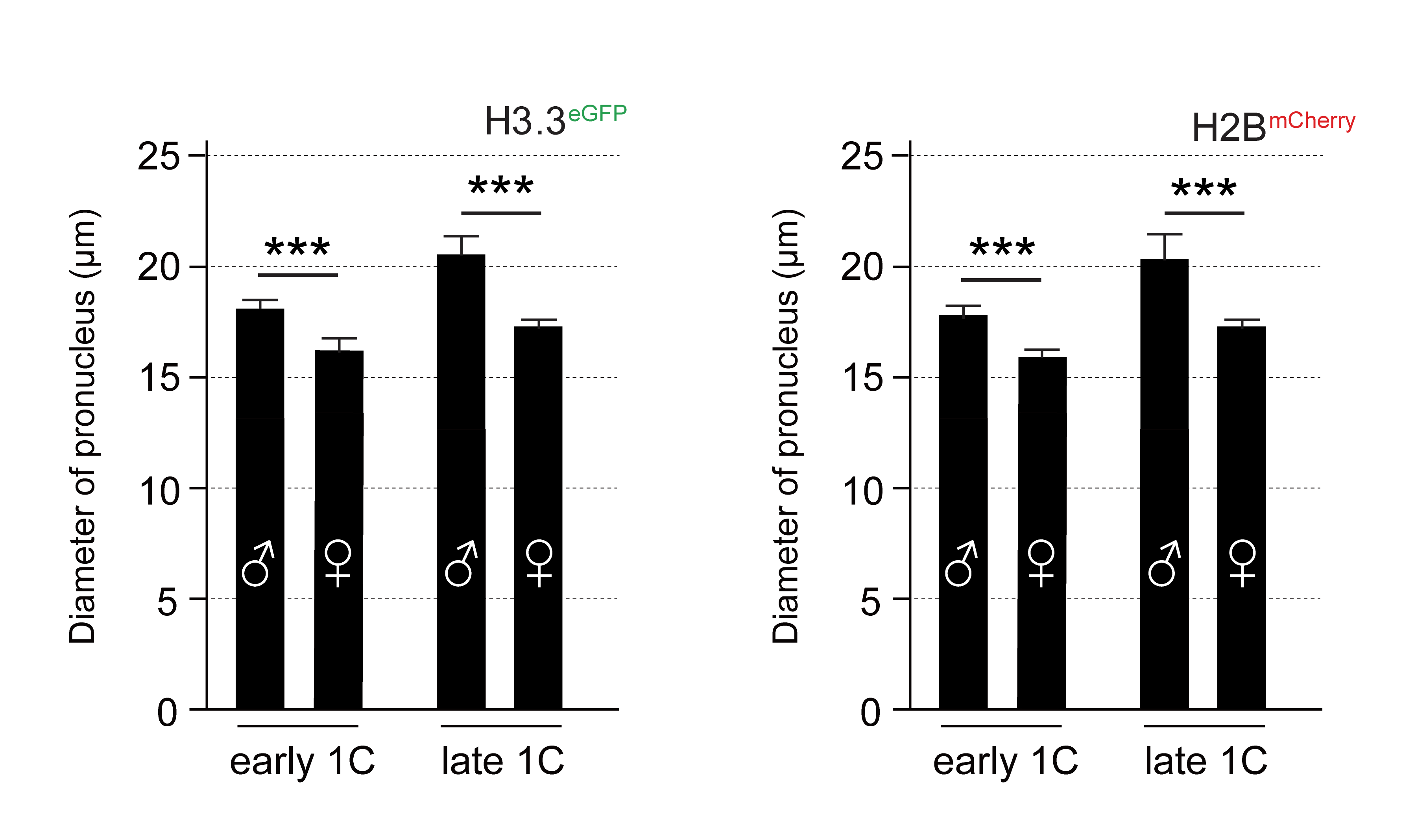

Supplement: Supplementary file 2 — Additional file 2: Figure S2. Quantification of diameters of male and female pronuclei in zygotes with maternally expressed H3.3-eGFP or H2B-mCherry. ***p<0.001 by Student’s t-test. [file 12958_2021_776_MOESM2_ESM.tif]
